# Indicators and criteria of consciousness: ethical implications for the care of behaviourally unresponsive patients

**DOI:** 10.1186/s12910-022-00770-3

**Published:** 2022-03-21

**Authors:** Michele Farisco, Cyriel Pennartz, Jitka Annen, Benedetta Cecconi, Kathinka Evers

**Affiliations:** 1 Centre for Research Ethics and Bioethics, Uppsala University, Uppsala, Sweden; 2 Science and Society Unit, Biogem, Biology and Molecular Genetics Research Institute, Ariano Irpino, AV Italy; 3 Department of Cognitive and Systems Neuroscience, Swammerdam Institute for Life Sciences, University of Amsterdam, Amsterdam, The Netherlands; 4 Research Priority Area, Brain and Cognition, University of Amsterdam, Amsterdam, The Netherlands; 5 Coma Science Group, GIGA-Consciousness, University of Liege, Liege, Belgium; 6 Centre du Cerveau, University Hospital of Liege, Liege, Belgium

**Keywords:** Consciousness, Disorders of consciousness, Neuroethics, Brain injury, Vegetative state, Unresponsive wakefulness syndrome, Minimally conscious state

## Abstract

**Background:**

Assessing consciousness in other subjects, particularly in non-verbal and behaviourally disabled subjects (e.g., patients with disorders of consciousness), is notoriously challenging but increasingly urgent. The high rate of misdiagnosis among disorders of consciousness raises the need for new perspectives in order to inspire new technical and clinical approaches.

**Main body:**

We take as a starting point a recently introduced list of operational indicators of consciousness that facilitates its recognition in challenging cases like non-human animals and Artificial Intelligence to explore their relevance to disorders of consciousness and their potential ethical impact on the diagnosis and healthcare of relevant patients. Indicators of consciousness mean particular capacities that can be deduced from observing the behaviour or cognitive performance of the subject in question (or from neural correlates of such performance) and that do not define a hard threshold in deciding about the presence of consciousness, but can be used to infer a graded measure based on the consistency amongst the different indicators. The indicators of consciousness under consideration offer a potential useful strategy for identifying and assessing residual consciousness in patients with disorders of consciousness, setting the theoretical stage for an operationalization and quantification of relevant brain activity.

**Conclusions:**

Our heuristic analysis supports the conclusion that the application of the identified indicators of consciousness to its disorders will likely inspire new strategies for assessing three very urgent issues: the misdiagnosis of disorders of consciousness; the need for a gold standard in detecting consciousness and diagnosing its disorders; and the need for a refined taxonomy of disorders of consciousness.

## Background

Consciousness, defined as subjective experience [1], can by definition be identified from a third-person perspective only through an inferential process: indeed, to assess consciousness in other subjects we have to rely on their subjective reports. When we cannot access subjective reports, such inferential character of consciousness assessment raises considerable challenges: how to diagnose consciousness in non-verbal or behaviourally non-communicative patients (e.g., patients with disorders of consciousness (DoCs)), who by definition cannot report on their subjective experience? Answering this question is notoriously challenging but increasingly urgent. The technological advancement in the detection of brain activity has resulted in new nosological categories and in new clinical classifications of patients with DoCs. In this paper we take as a starting point a recently introduced list of operational indicators of consciousness that facilitates its recognition in challenging cases like non-human animals and Artificial Intelligence (AI). While these indicators have been originally conceived to be applied to these cases, in this paper we investigate their relevance to patients with DoCs, whose high rate of misdiagnosis (i.e., due to difficulties in detecting their consciousness) is an urgent clinical issue with important ethical dimensions. On the basis of both clinical and ethical arguments, the assessment of consciousness in this clinical population is gradually moving from the behavioural standard (i.e., the Coma Recovery Scale revised, CRS-revised) towards integration with technological assessments (i.e., Electroencephalography (EEG)-based techniques, functional neuroimaging, and Brain-Computer Interfaces, BCI). Even though the use of neurotechnology in clinical settings is quite expensive and not always practically convenient, it is important to anticipate on further developments in this field [2–5] in order to complement behavioural diagnosis [6–8].

The aim of this paper is to show how the proposed indicators of consciousness, developed for the assessment of consciousness in AI and animals without or only very limited abilities for subjective report, can be useful for the assessment of consciousness in patients with DoCs. These patients have a compromised ability to communicate linguistically and behaviourally,^1^ which makes them unable or less able to reveal their conscious state to others, and raises the need for inferring their (residual) consciousness through relevant proxies. We here explore how objective indicators for consciousness (particularly their prospective operationalization) can contribute to the ethical discussion around DoCs, particularly concerning the clinical care of brain injured patients.

We start from a terminological clarification underscoring the importance of choosing the right words for referring to consciousness, the detection and knowledge of which, from a third person perspective, is necessarily indirect and inferential (i.e., hypothetical and tentative). This is particularly relevant to liminal cases like DoCs, where signs of consciousness may be flickering or almost imperceptible to an external observer (like in patients with cognitive-motor dissociation, CMD (see Table 1), who, according to neurophysiological assessments of residual cerebral activity, may be covertly conscious).

**Table 1:** Disorders of consciousness considered in the present paper (see [9, 10])

Vegetative state/Unresponsive wakefulness syndrome	Wakefulness accompanied by the absence of any sign of awareness
Minimally conscious state	Wakefulness accompanied by inconsistent but reproducible behavioural signs of awareness
Cognitive motor dissociation	Retained but unrecognized (covert) cognitive capacity for cerebral response to command in absence of purposeful behaviour

We then discuss the possible characteristics of residual consciousness in DoCs, which are hard to assess, but reasoning from the previously introduced indicators of consciousness, we can extrapolate which type of measurements and decoding approaches could work for patients with DoCs, stressing that their consciousness (if present) may be different from the one found in healthy conditions. On that basis, we analyse the possible ethical implications for DoCs of the suggested indicators of consciousness. With ‘indicators of consciousness’ we mean particular capacities that can be deduced from observing the behaviour or cognitive performance of the subject in question (or from neural correlates of such performance) and that do not define a hard threshold in deciding about the presence of consciousness, but can be used to infer a graded measure based on the consistency amongst the different indicators.

## Main text

### Words matter ethically: the inferential nature of the clinical detection of consciousness

Attributing consciousness to other subjects is *per force* inferential: the first-person experience is not shareable among different people because it is subjective in nature. In philosophy of mind this solipsism-like condition is expressed in the “other minds” problem.^2^ Inferring consciousness of other people is even more challenging with behaviourally non-communicative subjects (e.g., patients with DoCs) or subjects that are different from humans (e.g., animals and Artificial Intelligence (AI) systems). Whence the need for indicators for consciousness arises.

A set of such indicators has been recently introduced to facilitate the detection of consciousness in non-human agents [11], and these are relevant also to facilitating the assessment of consciousness in verbally or behaviourally non-communicative subjects, particularly in patients with DoCs.

Before focusing specifically on each indicator, a preliminary terminological clarification about them and the term they refer to (i.e., consciousness) is important. First, indicator is preferred to criterion for the following reason. The Merriam-Webster Dictionary defines a criterion as “a standard on which a judgment or decision may be based”, or also as “a characterizing mark or trait”. When applied to consciousness, the use of the term criterion might wrongly be taken suggest that: 1. a clear threshold can be detected in attributing/not attributing consciousness; 2. if that criterion is not satisfied, then consciousness is lacking. Given the inferential nature of attributing consciousness to others, both conclusions are fallacious. Particularly when DoCs are under scrutiny, we should bear in mind that absence of evidence is not *ipso facto* evidence of absence of consciousness [12, 13]. Also in the face of the high rate of misdiagnoses in this population, a cautionary approach is ethically and clinically justified [14].

Thus, the term “indicator”, with its less definitive and heuristic character (but also conceived to be applicable in practice), may be more appropriate than the term “criterion” in the concrete context of attribution of consciousness to others: the presence of an indicator suggests that consciousness is present; whereas the absence of an indicator does not rule out the possibility of undetected consciousness. In other words, that p–> q does not entail that -p – > -q.

The semantics of consciousness is among the most controversial issues in both science and philosophy: a wide array of conceptual and empirical models has been proposed, with controversies over how to define and how to measure it, as well as over its moral significance [15]. To assess such a panoply of meanings is beyond the scope and aim of this paper. The conceptual framework of the present analysis is represented by the view of consciousness as having a specific biological function, namely a modelling activity of the brain (i.e., a multimodal situational survey which takes the form of an inferential dynamic model or representation of the world) that basically enables to make complex decisions and to behave in order to get relevant goals, so that the subject can survive in its environment through the satisfaction of its needs and the achievement of its goals [16].^3^ Here, complex decision-making stands in contrast to reflexes and habits, which can be conducted largely unconsciously.

On the basis of such premises, what are the salient characteristics of consciousness? Pennartz, Farisco and Evers [11] have been identified the core features of consciousness summarized in Table 2,^4^:

1. *Qualitative richness*: conscious experience is qualified by distinct sensory modalities and submodalities (e.g. for vision, submodalities include texture, motion, colour, size, shape, depth);
2. *Situatedness*: consciousness is specified by the particular spatiotemporal condition of the subject, whose body occupies a particular place in space and time. Importantly, this concept includes objects with specific spatiotemporal relationships to each other (rather than departing from abstract space or time itself), as well as the subject’s body (as one object);
3. *Intentionality*: consciousness is about something other than its neuronal underpinnings;
4. *Integration*: the components of the conscious experience are perceived as a unified whole;
5. *Dynamics and stability*: conscious experiences include both dynamic changes and short-term stabilization.

**Table 2:** Key features of consciousness (= multimodal situational survey) in healthy subjects and in patients with Disorders of Consciousness

Feature	Description in healthy subjects	Description in patients with DoCs
Qualitative richness	Conscious experience is qualified by distinct sensory modalities and submodalities	Conscious contents (if any) might be limited in both sensory modalities and submodalities. They can be evaluated based on brain damage and residual behaviours (e.g. sniffing for smelling)
Situatedness	Conscious experience is specified by the subject´s spatiotemporal condition	Spatiotemporal framing, as well as bodily experience, might be changeable and discontinuous/fragmented
Intentionality	Consciousness is about something other than its neuronal underpinnings	Possible residual consciousness might be still intentional but less egocentric and more allocentric. Arguably decoding from the visual cortical system may indicate what residual visual experience is about
Integration	The components of the conscious experience are perceived as a unified whole	The elements of a scene might be perceived independently or at different levels of detail
Dynamics and stability	Conscious experiences include both dynamic changes and short-term stabilization	Being the anticorrelation between DMN and DAT compromised, residual conscious processing might be very unstable without any capacity for stabilization. Also the updating (dynamics) of conscious experience can be compromised

In short, the inferential model that consciousness provides to the subject is about inferred causes of sensory inputs (in line with Helmholtz and the principles of Predictive Coding, as applied to (conscious) perception [16, 18]). However, drawing an inference does not make the representation conscious per se, because it needs to be sufficiently “big” and comprehensive (i.e., multimodal and situational) to qualify as being conscious [19]. Moreover, sensory inference in healthy subjects only qualifies as conscious when it is intentional, integrated, and alternately dynamic and stable, in addition to being qualitatively rich and spatiotemporally situated.

Do these characteristics of consciousness change in the case of compromised consciousness, like in patients with DoCs, and if so, how?

### Residual consciousness in patients with DoCs

We are aware that the very concept of levels or grades of consciousness is controversial: some scholars argue that consciousness is an on/off (all or nothing) rather than a gradable phenomenon [20, 21]. Notwithstanding this controversy, the differentiation between levels and contents of consciousness, roughly corresponding to wakefulness and awareness respectively, is widely used in the clinical assessment of consciousness, particularly in cases of DoCs [22], where it is assumed that a subject might be conscious at different levels of intensity.

Besides this practical reason to refer to different levels of consciousness, there is also a “common sense” intuition that we can be conscious at different levels, which can be understood, for instance, as intensity (e.g., intense pain rather that barely noticeable), complexity (information content) in one modality and multimodal richness (different qualia).

This differentiation between different levels of consciousness seems to be valid both intra-personally (i.e., an individual experiences different levels of consciousness at different points in time) and inter-personally (i.e., we sometimes have the intuition of experiencing different levels of consciousness compared to others, including non-human beings [23, 24], even if we are not able to specifically assess their level of consciousness).

That said, how might we characterize (residual) consciousness in patients with DoCs? If it is true that characterizing consciousness in other people is challenging, it is even more so for non-verbal or behaviourally impaired people like patients with DoCs. Such conjectures are nevertheless justified on the basis of an inference to the best explanation: we are not sure about the characteristics of the consciousness of such people, but given our current understanding of consciousness it is reasonable to infer some relevant qualities. Furthermore, we can assess the patient’s brain state—whether this is closer to wakefulness than to e.g. anaesthesia or slow-wave sleep, and we can also attempt to decode from brain activity what information is still processed.

Specifically, for each of the features of consciousness identified above, it is possible to hypothesize how it is affected and eventually re-shaped in patients with DoCs (see Table 2).

1. *Qualitative richness*: conscious contents (if any) are arguably limited with regard to both sensory modalities and submodalities and central neural correlates. To illustrate, patients with DoCs might be unable or only partially able to experience e.g. vision, sound, smell, taste, touch, pleasure or pain.
  How much qualitative richness is compromised depends on the extent to which the relevant neuronal structures are damaged. This might also be tested studying residual behavioural responses (e.g., sniffing for smell) and technologically assessing anatomical brain damage or loss of physiological function, pointing to loss of a particular sense, taking into account the possibility that the brain may be plastically reorganized, with resulting (partial) recovery of the sense. This possibility can be assessed using brain imaging (e.g., functional Magnetic Resonance Imaging (fMRI), Positron Emission Tomography (PET) or Diffusion Tensor Imaging (DTI)). Recent studies have showed that late recovery related to brain plasticity (e.g., axonal regrowth) is more frequent than previously thought [25, 26]. This highlights the fact that all the possible assessments of consciousness in patients with DoCs (including our indicators) target actual consciousness while they do not give information about possible future recovery.
2. *Situatedness*: spatiotemporal framing might be changeable and discontinuous, like in dreaming experience or altered states of consciousness, e.g. autoscopy (seeing yourself at a different place than where your body is), out-of-body experience (experiencing the world from a location outside your body), type II blindsight (having a feeling that a change occurred within your blind area, that feeling not being a visual percept), loss of selfhood, ketamine effects. Also, bodily experience might be reframed by brain impairment, and this might impact residual consciousness.
3. *Intentionality*: the aboutness of possible residual conscious experiences might be preserved, though possibly disconnected from a stable ability to refer those experiences to a self. Consciousness might be less egocentric and more allocentric than in healthy subjects. Ego-centricity and allo-centricity are here used not in an object-oriented meaning (i.e., self-consciousness vs externally oriented consciousness), but rather in an action-oriented meaning (i.e., the capacity to model the world starting from a stable sense of self vs the capacity to model the world exposed to (more or less) random external modulation or starting from an unstable or “flickering” sense of self). In fact, in DoCs selfhood (including proprioception, sense of balance, etc.) might be unstable while the brain might partially keep its ability to model the world.
4. *Integration*: the wholeness of a conscious percept might be compromised, and the different elements of a scene eventually perceived independently or at different levels of detail. Among the relevant neurological conditions are: hemineglect (failing to be aware of items to one side of space); scotoma (an area of partial alteration in the visual field which results in a partially diminished/entirely degenerated visual acuity surrounded by a field of normal vision), simultagnosia (inability to perceive more than a single object at a time); visual agnosia (apperceptive: seeing lines but not objects).
5. *Dynamics and stability*: residual conscious processing might be very unstable without any capacity for stabilization (while normal consciousness, even if dynamically evolving and thus “unstable”, might usually be stabilized at will). Relevant illustrations of this impaired dynamics and stability come from Parkinsonism where the dynamics of perception may be compromised (frame-by-frame views [27]). Relevant results derive from a recent study on the temporal circuit subserving consciousness in the brain, specifically the anticorrelation between the Default Mode Network (DMN) and the Dorsal Attention Network (DAT), cortical networks correlated to internal and external orientation respectively: the disruption of this temporal circuit affects the subjective capacity for stable perception and appears to be a common signature of unresponsiveness of diverse aetiologies [28, 29].

Furthermore, both the amount and the dynamics of sensory inputs are drastically reduced in DoCs, the patient being lying in a bed with impaired sensory abilities.

### Ethical implications of indicators of consciousness

As mentioned above, indicators of consciousness are operational features which can be detected and assessed from external observations and that in combination with each other can serve as an overall criterion for attributing consciousness.

Before reflecting on the specific ethical relevance of each indicator to the diagnosis and potentially to the treatment of patients with DoCs as illustrated in Table 3, it is useful to outline some general points shared by all of them.

**Table 3:** Indicators of consciousness and respective ethical implications in disorders of consciousness

Indicator of consciousness	Description	Ethical implications in DoCs
Goal-directed behavior (GDB) and model-based learning	GDB is a behaviour aimed at achieving specific goals on the basis of two essential conditions: the ability to represent the consequences of subjective actions; the knowledge that those particular actions are instrumental for achieving desired goals. Model-based learning is defined as the capacity for an internal model of the subjective spatiotemporal condition, including particular connections between automatic and voluntary behaviours and their outcome	GDB and model-based learning are indicative of the ability to have conscious interests, to recognize the relevance of external inputs to fulfill those interests, and to act on the basis of those inputs for fulfilling interests. Checking for their presence is ethically required, possibly through a multimodal assessment
Brain anatomy and physiology	In mammals, consciousness depends on the structural and functional integrity of specific anatomic structures in the brain with a characteristic physiology. Similar brain structures indicate the presence of consciousness	In the case of DoCs, structural and functional damages of thalamocortical systems impair consciousness. The main ethical issue is whether consciousness is switched off or is still present to a limited degree. Caution in inferring absence of consciousness from brain damage is recommended
Psychometrics and meta-cognitive judgment	Psychometric curves for stimulus detection and discrimination, as well as the ability of some meta-cognitive judgments on perceived stimuli, show similarities between humans and some animals (e.g., monkeys, rodents, and birds). The same is arguably true for patients with DoCs: if behavioural or instrumental tests show psychometric curves similar to healthy subjects, consciousness might be inferred	Stimulus detection and relative meta-cognitive judgment are evidence of retained awareness in DoCs. Exploring strategies for detecting them, both through behavioural tests and instrumental decoding of cortical activity, is ethically required
Episodic memory	This type of memory is defined as autobiographical or narrative memory, i.e. memory of facts (‘‘what’’) that are spatiotemporally specified, i.e. experienced at a particular place (‘‘where’’) and time (‘‘when’’)	Exploring strategies for assessing relevant neuronal structures, looking for correlates of episodic recall (e.g., relevant hippocampal regions), is ethically required
Acting out one’s subjective, situational survey: illusion and multistable perception	Studies with non-human primates and cats showed their susceptibility to illusions and perceptual ambiguity, and there are evidences for rodents and birds as well	Exploring possible strategies for assessing relevant neuronal structures is ethically required. If the patient is behaviourally unresponsive, autonomous indicators (e.g., electrodermal activity, galvanic skin response, pupils) or stimuli reactions in relevant brain regions might be considered
Acting out one’s subjective, situational survey: visuospatial behaviour	Conscious subjects perceive objects as stably situated, even when they explore their environment with their gaze	Its relevance to patients with DoCs seems limited (if at all relevant) unless we look for replacement measures: no overt behaviour but decoding of internal brain activity indicating the use of one’s body map and the planning of a spatial response

First, as already mentioned, rather than providing definitive evidence of consciousness, indicators should be taken as indicative and provisional, but are conceived to be applicable in practice. This means two things in particular: 1. They may be used to support an ethical decision, but they are insufficient in themselves to justify it: further arguments are needed, both from other indicators and from other kinds of resources, like ethical and philosophical arguments as well as the patient’s (anticipated) will/ advanced directive. This is consistent with the rationale behind the indicators of consciousness, i.e. evidence of consciousness optimally accumulates across the assessment of multiple indicators, which can be summed up to yield an overall scale of the score. 2. The absence of a positive indicator does not exclude consciousness. From a clinical/ethical point of view, this suggests precaution, especially in order to avoid considering unconscious someone who is actually conscious. This is particularly applicable to patients with DoCs, whose brains have undergone massive structural and functional damage and possibly consequent re-organization, which entails that a covert form of consciousness might be retained even if in the absence of relevant indicators. The fact that absence of evidence (from indicators) does not imply evidence of absence (of consciousness) does not entail that the indicators are irrelevant or useless from an ethical point of view. Even if not conclusive, they can still facilitate the detection of residual consciousness, and even minimal evidence for it might make a significant difference in terms of treatment. To illustrate this point, the mere abstract possibility that a patient may retain consciousness in the absence of substantial evidence might be insufficient for justifying administering painkillers, because of their possible side-effect (e.g., reduction of residual consciousness [30]), and here indicators of consciousness may help in providing more elements for a balanced decision. In other words, they do not solve uncertainty completely, but can help to reduce it significantly.

Addressing the inferential character of consciousness assessment and the problems arising from it, is widely recognized as an ethical priority in the clinical treatment of patients with DoCs [31]. Notably, appropriate protocols for conducting this kind of assessment (especially for adequately communicating with the patient´s family members) should be further developed. To illustrate, the Perturbational Complexity Index (PCI) has been introduced as a theory-based index of consciousness independent of processing of external sensory inputs and behaviour [32, 33]. That index is based on the assumption that consciousness depends on the brain’s ability to support complex activity patterns distributed among interacting cortical areas and differentiated in space and time [34, 35]. It is evaluated by Transcranial Magnetic Stimulation (TMS) of the cortex and measuring the informational complexity of the pattern of the evoked EEG responses [33]. Among other measures, the PCI might be used to refine the classification of patients with DoCs [36]. The Bispectral Index (BIS) is another EEG-based index used to monitor levels of consciousness in clinical settings, particularly in anesthetized patients. Its diagnostic and prognostic use in DoCs has been investigated, showing promising results compared to other EEG-based methods [37, 38]. The refinement and further development of these and other assessment protocols should be complemented with a dedicated focus on the need to translate the data they provide in effective communication procedures trying to adequately inform the persons who should make decisions regarding the patient’s treatment, (i.e., medical doctors, family members and/or legal guardians).

Second, the likelihood of consciousness will increase if more indicators are found positive simultaneously [11]. It is therefore ethically important to assess most if not all of them in controversial cases like patients with DoCs. This would imply using all the available tools, both behavioural (e.g., the CRS-revised) [39] and neurophysiological (based on e.g. EEG, functional neuroimaging, spike data), to make such an assessment. This last point is in line with recent guidelines published by the European Academy of Neurology (EAN) [6] and the American Academy of Neurology (AAN) in collaboration with the American Congress of Rehabilitation Medicine (ACRM) and the National Institute on Disability, Independent Living, and Rehabilitation Research (NIDILRR) [8]: both recommend the multimodal assessment of consciousness integrating behavioural, EEG-based and neuroimaging-based measures. Notably, the non-invasive EEG approach may be extended to invasive Electrocorticography (ECoG) and/or high-density, multi-neuron spike recordings, allowing more refined and detailed assessments, including targeting of consciously represented content—going beyond the mere assessment of brain state [2, 3].

The EAN document outlines the immediate clinical impact that low-cost and easy-to-implement bedside measures can have (e.g., probing for voluntary eye movements using a mirror, relying on repeated clinical assessments, favouring the Full Outline of Unresponsiveness (FOUR) score over the Glasgow Recovery Scale (GRS) in acute settings, and clinical visual analysis of standard EEG). Continuing on the point of innovative neurophysiological techniques (e.g., high-density EEG, ECoG and parallel, multi-neuron spike recordings), even if logistically challenging and requiring more expertise, these may enable a more refined evaluation of residual consciousness, including the detection of covert consciousness. Given the high rate of misdiagnosis still affecting the assessment of patients with DoCs [40, 41], this refined evaluation is urgently needed, because looking only at the brain state may not be sufficient for assessing consciousness and is preferable to decode the content of experience. Relevant results in this direction have been obtained through neural decoding of visual imagery during dream sleep [42]. A recent study by Naci et al. seems also very promising in this respect [43]. Using naturalistic paradigms (e.g., watching a movie or listening to an audio–video), the authors first suggested, using a combination of fMRI data and statistical parametric modeling, that a common neural code likely supports conscious experience in healthy people. On that basis, the authors then continued to gather evidence that the same neural code can be used to interpret conscious experience in patients with DoCs, not using an active paradigm (i.e., instructing the patient to do particular tasks), but rather engaging his attention through meaningful stimuli that are similar to real-world sensory information. Particularly, showing a Hitchcock movie to two behaviourally unresponsive patients with an unknown level of consciousness, they found that activity in a network of frontal and parietal regions which support executive processing was significantly correlated to the EEG/fMRI pattern of healthy people. As the authors acknowledge, this is not sufficient to figure out the details of the patient’s thoughts, but it is a promising strategy to detect the kind of executive processing thought to be associated with conscious experience.

Thus, the need for integrating behavioural and neural assessments of residual consciousness in patients with DoCs can be justified from different points of view. Like the EAN, also the AAN, ACRM, and NIDILRR document, after having repeatedly recommended the use of serial standardized neurobehavioural assessments, advises the use of multimodal evaluations including functional neuroimaging or electrophysiology studies when neurobehavioral assessment is ambiguous or confounders to a valid clinical assessment are identified.

The abovementioned indicators might require using this kind of multimodal approach while at the same time helping in defining a practical strategy to implement it.

### Indicator 1: goal-directed behaviour and model-based learning

Goal-directed behaviour (GDB) can be described as a behaviour aimed at achieving specific goals on the basis of two essential conditions: the ability to represent the consequences of subjective actions; the knowledge that those particular actions are instrumental for achieving desired goals [44, 45]. Command following as behaviourally assessed in DoC patient is not necessarily the same as GDB: although usually interpreted as intentional and voluntary, it could rely solely on stimulus–response reactions.

The concept of model-based learning related to GDB can be defined as the capacity for an internal model of the subjective spatiotemporal condition, including particular connections between automatic and voluntary behaviours and their outcome [46]. These two concepts are related but diverge in some respects: whereas GDB emphasizes both the fact that the subject is aware of the connection between his action and related outcome and that this connection is contingent, model-based learning focuses specifically on the capacity to model the connection between stimulus, action, and related outcome, on the capacity to anticipate future occurrences, and on the capacity of real-time and spontaneous decisions [11, 46, 47].

Even though their relationship with consciousness is not straightforward, GDB and model-based learning can arguably indicate it, because they both require much more than reflexes and behaviour that has become automated through habit formation. The rationale is that conscious experience facilitates or enables GDB: in order to make temporally deep plans, subjects need a multimodal survey of their environmental and bodily situation [48]. Moreover, both GDB and model-based learning indicate the ability to have interests, to recognize the relevance of external inputs to fulfil those interests, and to act on the basis of those inputs for fulfilling interests. Even though these latter factors point more to motivation than consciousness, these are all ethically relevant abilities: when detectable in patients, they call for an ethical evaluation, which means that assessing whether they are present is ethically required when making decisions about care, treatment, diagnosis, and prognosis. In the case of DoCs, these abilities might be covert and flickering (i.e., not detectable at the bedside and inconsistent in time). This seems to be the case in patients with CMD (i.e., able to follow commands by medical doctors of imagining to move their body while their brains are monitored through recordings (e.g. fMRI and EEG), even if unresponsive at the bedside)[10]. Some promising results from which one may infer residual capacities for GDB and model-based learning in patients with DoCs emerged from the use of fMRI [49, 50]: some patients were able to modulate their brain activity by generating blood-oxygenation-level-dependent (BOLD) responses that were judged by the researchers to be induced voluntarily, reliably and repeatably. With specific reference to GDB, a test for prospective path planning has been done in healthy humans showing neural computations underlying our ability to make fast and robust multi-step inferences in the absence of prior learning, with a critical role played by the Hippocampus coupled with rostrodorsal medial prefrontal cortex (rd-mPFC)[51]. It will be both scientifically and ethically interesting, as a concrete way to advance the detection of residual consciousness, to expose CMD and other patients with DoCs to this task or a reduced version of it in order to get further information about their residual brain activity likely indicative of residual consciousness, especially if there is no overt behaviour.

The wilful modulation of brain activity detected through fMRI in experimental settings, particularly when consistent with external requests by the experimenters, may yield evidence of retained GDB and model-based learning. This is another reason for calling for an increasing inclusion of technological diagnostic tools in clinical practice, as argued also by Peterson et al. [52]. The main ethical and clinical reason for the use of neurophysiological assessment of residual consciousness in DoCs is that the behavioural standard has shown limited reliability [14, 53]. But the clinical usefulness of diagnostic neurotechnology is even more justified if it can help to detect ethically relevant abilities like GDB and model-based learning. Notwithstanding a type-correlation between neuronal and mental activities (i.e., we can infer that mental activity is going on the basis of the neuronal activity we detect), a token-gap still exists between them (i.e., we are not yet able to identify a specific conscious experience correlated to a particular pattern of neuronal activity) (Naci et al. [43], even if some empirical attempts have been made in this direction [42, 54].

In conclusion, GDB and model-based learning as joint indicator of consciousness imply the ethical need to check for residual relevant cognitive abilities (in contrast with the execution of reflexes or simple habits) in patients with DoCs, requiring complementation of behavioural with technological assessments. To the extent that these abilities are detected, they require an ethical evaluation, for both formulating the best possible diagnosis and planning the most appropriate treatment.

### Indicator 2: brain anatomy and physiology

The rationale behind this indicator of consciousness is that, in mammals,^5^ consciousness depends on the structural and functional integrity of specific anatomic structures in the brain with a characteristic physiology, so that their presence in other mammals (or the presence of resembling structures in other species) can be taken to indicate consciousness.

Applied to DoCs, this indicator suggests focusing on so called neural correlates of consciousness (NCC) [58, 59] and to check their integrity. More specifically, a NCC can refer to a general, global state of consciousness (as neural correlates that mark the difference between being and not being conscious)[60, 61], or to particular contents of consciousness (as neural correlates that are sufficient for being conscious of a specific object or scene) [62, 63]. Regarding content-specific NCCs for vision, there has been a debate whether to identify them with systems in the prefrontal cortex (with late activations to reported stimuli) or with systems in occipital/parietal cortices (showing early activations) [63]. The increasingly accepted view is that the latter hypothesis is the more likely, while late activation in prefrontal cortex would be a correlate of metacognition, attention, task execution, working memory and behavioural reporting rather than of consciousness [16, 64–66]. Accordingly, damage to the prefrontal cortex does not cause loss of consciousness, except for the orbitofrontal cortex in the right hemisphere (loss of smell; [67, 68]).

The same holds for NCCs of state consciousness. Even in this case the best current anatomical candidates for conscious vision are localized in a temporo-parietal-occipital zone of the posterior cerebral cortex [65].

As mentioned above, in clinical studies of DoCs two components of consciousness are usually identified: wakefulness and awareness. Their respective correlates have been investigated. The functional and structural integrity of ascending ponto-mesodiencephalic reticular pathways and widespread thalamocortical projections has been shown to be essential for igniting and maintaining the level of consciousness (i.e., wakefulness) [69, 70] even though no correlation between thalamic atrophy and arousal has been found in patients with DOCs [71].

Starting from the clinical/operational distinction between two components of consciousness, i.e. level (wakefulness) and content (awareness)[22], it has been reported that, besides the activation of low-level specialized cortices [72], awareness requires the activation of a wide brain network, including parietal cortex (including parieto-temporal and posterior parietal areas bilaterally) and, in case of attempts to follow commands behaviourally, frontal regions [73], even if cortical activity per se is not sufficient for conscious processing of information (e.g., if not sufficiently inter-connected to other cortical areas to lead to a global ignition [74, 75].

DoCs are caused by traumatic or non-traumatic brain injuries, i.e. structural and functional damages of thalamocortical systems, which impair consciousness [22, 76, 77]. Thus patients with DoCs have both brain anatomy and physiology variably impaired. What does it mean in terms of possible residual consciousness? The main ethical issue arising here is whether consciousness is switched off in the lowest level of DoCs or is more gradually lost and possibly still partially present despite brain damage: can we infer from the impairment of brain structures and functions that relevant cognitive functions are impaired and/or lost? And what does this mean for possible residual consciousness? Dissociation between residual cognitive abilities and consciousness might exist in patients with DoCs, who may fail tests for consciousness not because they are unconscious but because they are unable to perceive stimuli in particular modalities or cognitively process them [41, 78].

### Indicator 3: psychometrics and meta-cognitive judgment

Psychometric curves for sensory detection and discrimination are already assessed in patients with DoCs (e.g., through the Disorders of Consciousness Scale, DOCS, a bedside test evaluating recovery of neurobehavioural functions [79, 80], with an open discussion as regards the possibility to improve it [81]). For instance, bedside assessment might be complemented by recording neuronal activity in a relevant area (with/close to NCC) and making a neurometric curve, possibly with an additional measure of consciousness (e.g., heartbeat, optokinetic response, etc.).

Different experimental tests (e.g., orientation to self, orientation to environment, auditory, tactile, and noxious items) have been introduced in the bedside assessment procedures like the Coma Recovery Scale-Revised[39], with related test items, administrative procedures, expected response modes, and scoring examples. Indeed, in general a high score on this kind of test is a strong indicator of preserved conscious activity. The problem with administering these tests to patients with DoCs is that their underlying cognitive abilities might be 1. too compromised to allow an understanding of the questions raised by the clinical staff, or 2. flickering or absent when the test is administered, despite a residual conscious activity. Regarding point 1, a possible solution might be the use of some tests that seem less sensitive to language understanding and that are more involving, such as visual pursuit with a mirror (the self-referential aspect seems to engage patients more). Also, the assessment of aphasia might help to reduce behavioral clinical misdiagnosis. [82].

An alternative for improving the detection of residual conscious processing through the quantification of psychometric curves might be, for instance, to decode stimulus detection from residual neural activity in relevant cortical areas, complementing the behavioural assessment with mechanistic investigation. Importantly, the decoded neural activity should closely reflect the psychometric responses as recorded in healthy persons, otherwise uncertainty about the status of consciousness increases.

It seems even harder to assess meta-cognitive judgment ability in these patients and to use it as a ground for detecting residual consciousness. We refer to metacognitive judgement in the context of perception, stimulus valuation and consciousness, basically as confidence judgement and post-decision wagering [83, 84]. In fact the usual way of assessing meta-cognition is through verbal reports, even if some non-verbal tests have been introduced in a comparative metacognition assessment [85]. For instance, in animals, response latency has been used (where longer latency correlates with more uncertainty), but a caveat in this measure is that other factors, unrelated to consciousness (e.g., automated motor preparation) can come into play here. A stronger process to consider in this context may be vicarious trial and error-type of behaviour and its neural correlates, which can be recorded for example from hippocampus [86, 87].

### Indicator 4: episodic memory

Episodic memory is circumscribed as autobiographical or narrative memory, i.e. memory of facts (‘‘what’’) that are spatiotemporally specified, i.e. experienced at a particular place (‘‘where’’) and time (‘‘when’’). In humans, episodic memory is consciously recalled by definition and can be verbally reported about. Episodic recall is also closely associated with the conscious experience of an event before this is stored in declarative memory, so that episodic memory (which, together with semantic memory, constitutes declarative memory) is arguably an indicator of consciousness.

Relevant brain structures and functional networks (e.g., neocortical areas and hippocampus) should be interrogated in order to check whether residual episodic memories can still be consciously recalled in DoCs and/or reported. Also regarding the assessment of episodic memories in DoCs, there is still an open gap between the detection of neuronal activity and deciphering the particular content of related mental activity, relevant promising empirical results notwithstanding [88, 89]. One approach is to wait until recovery of the patient, and let him/her report afterwards what was experienced [90], but this would be useless when, for instance, particular clinical decisions should be made before possible recovery. Another approach is to be less ambitious than decoding experienced content, but rather to search for neural markers indicative of episodic memory retrieval (e.g., hippocampal spike sequences organized by the theta rhythm in the case of wakeful, prospective processing [91, 92].

Another possible strategy for detecting episodic memory is neural assessment of relevant brain functions. Yet even in this case we encounter the problem of inferring specific mental abilities from assessed neuronal functions. For instance, replay processes are probably not coupled to consciousness. On the other hand, hippocampal recordings [93] showed neural correlates of conscious memory recall (e.g., of Homer Simpson movies) in epileptic patients. Thus, in principle neural correlates of episodic memory recall may be used as an indicator of consciousness in DoC patients.

### Indicator 5: acting out one’s subjective, situational survey: susceptibility to illusions, multistable perception and visuospatial behaviour

The indicator of visuospatial behaviour relies on the ability to perceive the external environment and to act on this perception by expressing visuospatial behaviours that require the presence of a multimodal, situational survey. They presuppose the fundamental ability of the conscious subject to have a multimodal, spatiotemporally ordered perception of its environment and to behave accordingly within it. These behavioural abilities are obviously heavily compromised in patients with DoCs, and consequently the relevance of the corresponding indicator is limited. Particularly, Vegetative State/Unresponsive Wakefulness Syndrome (VS/UWS) patients show no behavioural evidence of environmental awareness, while Minimally Conscious State (MCS) patients can exhibit consistent command following as well as purposeful behaviours like intentional object tracking [94].

Nevertheless, the case of subjects with CMD and covert awareness shows that environmental perception might be dissociated from visuospatial behaviour, and that in patients with DoCs the latter is not applicable to probing retained consciousness. But again, one can think of several replacement measures that assess internal brain function. Instead of actual visuospatial behaviour, one may record neural activity correlating to path planning in space (cf. [95]).

As regards the indicator on illusions and multistable perception, it is worth investigating whether seeing an illusion (e.g., rotating snakes; [96]) elicits different emotional and arousal responses in DoC patients than not seeing an illusion (i.e., a more neutral picture). When presenting pictures eliciting salient, arousing illusions it will be relevant to assess heart rate, eye movement, and pupil size: for instance, eye movements can sometimes indicate what people are seeing and tracking (e.g,. nystagmus in a no-report paradigm; [66]). If possible, neural correlates of illusory perception may be recorded from patients, such as those expressed in top-down influences from higher visual to primary visual areas in the case of visual illusory contour perception (e.g., Kanizsa triangle; [97, 98].

Thus, where overt visuospatial behaviour or overt responses to illusions are lacking, we need to consider derived (extrapolated) measures to assess these indicators in patients with DoCs.

## Discussion

Different questions arise about the proposed application of the indicators of consciousness described above to DoCs. Among them, why are these indicators relevant to DoCs? What is the rationale to apply these indicators to DoCs? What would be new in this approach?

Concerning the relevance issue, these indicators were initially conceived to be testable by external observation of non-verbal subjects (i.e., animals)^6^ that cannot overtly (i.e., linguistically) prove their state of consciousness, which must thus be inferred from proxies. Patients with DoCs are in a very similar condition, possibly even worse: they cannot linguistically present evidence of their consciousness and in some cases they cannot behaviourally manifest it either, or they retain a very limited set of behavioural abilities. We might say that in order to assess consciousness in patients with DoCs the need for inferential reasoning is even bigger than in animals. For this reason indicators for consciousness are relevant also to patients with DoCs. However, when patients cannot display behavioural responses, it is mandatory to seek equivalent or derived cerebral or bodily measures of these indicators. Along this line, we propose some practical directions and tests that can be carried out to assess DoCs and quantify residual consciousness better. To illustrate, GDB might be investigated in patients with DoCs adapting a test for prospective path planning previously developed for healthy subjects. Brain anatomy and physiology might be operationalized in terms of NCCs. Psychometrics and meta-cognitive judgment might be assessed in patients with DoCs decoding stimulus detection from residual neural activity in relevant cortical areas and comparing it with the psychometric responses of healthy subjects, and using vicarious trial and error-type of behaviour and its neural correlates respectively. Episodic memory might be assessed in patients with DoCs searching for neural markers indicative of episodic memory retrieval. Finally, as an indicator of consciousness, the susceptibility to illusions, multistable perception and visuospatial behaviuor may be assessed in patients with DoCs recording neural activity correlating with path planning in space, and with visual illusions, as well as assessing derived measures like relevant physiological data (e.g., heart rate, eye movement, and pupil size).

It is important to keep in mind that the indicators in question do not reveal directly whether the subject under scrutiny has rich phenomenal experience or retains intentionality, because they target cognitive mechanisms closely linked to those underlying consciousness. Apart from targeting such closely related mechanisms (such as underlying goal-directed behaviour), it has been argued that phenomenal consciousness and its underlying computational mechanisms can be conceived as occurring at a different level of the same representational capacity [16]. Once we understand this relationship better, it may become more feasible to address the neural correlates of consciousness more directly, eventually also in DoC patients. In fact, the problem of how to target the first-person experience more directly stands in need of further analysis.

The application of these indicators to DoCs can be justified along three lines of argument concerning three very urgent issues: the misdiagnosis of DoCs; the need for a gold standard in detecting consciousness and diagnosing its disorders; and the need for a refined taxonomy of DoCs. These three paths provide also a justification of the ethical relevance of the indicators.

As mentioned above, one of the main problems affecting the assessment of residual consciousness and consequently the healthcare of people with DoCs is a high rate of misdiagnosis, specifically the difficulty in disentangling the different types of DoCs. This mainly depends on the fact that the standard protocols used in clinical settings are behavioural, i.e. relying on observable patients’ reactions to a number of different external stimuli. Obtained results are then aggregated, and the patient classified according to the deriving index. Among many possible shortcomings, this approach fails to detect potential covert forms of consciousness that might be retained by the patients. For this reason an instrumental assessment (functional neuroimaging, EEG-, ECoG or ensemble-spike based) of residual cognitive abilities has been introduced (first in research settings and prospectively in clinical practice), both based on wilful modulation of brain activity in response to external instructions (e.g., verbal commands by the experimenter) or on brain modulation in reaction to relevant environmental stimuli (e.g., watching a movie).

As many relevant papers and guidelines recommend, the integration of behavioural and instrumental assessments would be an ideal strategy to implement. To illustrate, the EAN document explicitly states that standardized clinical rating scales (e.g., CRS-R and FOUR), EEG-based techniques and functional neuroimaging (fMRI and PET) should be integrated into a composite reference standard [6]. While this recommendation is agreeable in principle, the question how to implement it in practice remains open. What seems crucial is to operationalize consciousness measures. The indicators of consciousness are conceived exactly to set the theoretical stage for an operationalization of consciousness, i.e. to develop an operational concept suggesting a more comprehensive strategy of how to measure consciousness and then to make it measurable, as well as a testable set of abilities that can be checked in patients with DoCs. In this way, the indicators may help to practically implement the recommended integration between these two assessments offering a general, overarching theoretical framework. While this paper is limited to the theoretical side of the issue, it might inspire further empirical attempts to operationalize the identified indicators of consciousness.

In clinical context, the so-called “gold standard” is conceived as the condition with the highest validity, i.e. the highest correspondence with what is under scrutiny [99]. With respect to the diagnosis of DoCs, the gold standard is the population of subjects on which the consciousness metric should be validated [100]. In other words, the gold standard is a kind of paradigm against which the particular case of the patient in question is evaluated. In the case of consciousness assessment, if the healthy condition is assumed as paradigmatic, or the metric for consciousness is calibrated on a healthy population, the problem of translating it to patients with DoCs arises. This problem derives from the fundamental fact that there is no consensus on the nature of consciousness nor on the essential measurable phenomenon that contributes to its realization [99]. The indicators of consciousness might help in overcoming this challenge focusing on quantifiable cognitive abilities which, as we have argued, can be considered proxies for consciousness in both healthy subjects and patients with DoCs.

The actual taxonomy of DoCs has been criticized because dichotomic, binary distinctions are unable to account for the more graded condition characterizing the affected patients, whose consciousness is not disordered in exactly the same way for everyone with the same diagnosis (i.e., VS/UWS or MCS). A multidimensional account of consciousness focused on different relevant abilities (i.e., semantic comprehension, attentional control, speech production, volitional control, visual tracking and fixation, executive control, metacognition, global incongruency detection) has been suggested as a ground for rethinking a taxonomy of DoCs accounting for their complex condition [21]. Indicators of consciousness might complement this attempt to reform the nosology of DoCs, helping both to refine the list of relevant cognitive dimensions in a theoretically grounded fashion and to set up strategies for detecting them in patients with DoCs.

## Conclusions

The indicators of consciousness described above are relevant for assessing residual consciousness in DoCs and for this reason they raise specific ethical implications. They indicate new theoretical perspectives that can inspire new strategies for operationalizing and quantifying relevant cognitive and cerebral functions. Particularly the indicators of consciousness analysed in this paper can fruitfully contribute to assess three very urgent clinical and ethical issues: the misdiagnosis of disorders of consciousness; the need for a gold standard in detecting consciousness and diagnosing its disorders; and the need for a refined taxonomy of disorders of consciousness.

## Data Availability

All data generated or analysed during this study are included in this published article.

## Abbreviations

AAN: American Academy of Neurology
ACRM: American Congress of Rehabilitation Medicine
AI: Artificial intelligence
BCI: Brain–computer interfaces
BIS: Bispectral index
BOLD: Blood-oxygenation-level-dependent
CMD: Cognitive-motor dissociation
CRS-revised: Coma recovery scale revised
DAT: Dorsal attention network
DMN: Default mode network
DoCs: Disorders of consciousness
DOCS: Disorders of consciousness scale
DTI: Diffusion tensor imaging
EAN: European Academy of Neurology
ECoG: Electrocorticography
EEG: Electroencephalography
fMRI: Functional magnetic resonance
FOUR: Full outline of unresponsiveness
GDB: Goal-directed behaviour
GRS: Glasgow recovery scale
MCS: Minimally conscious state
NCC: Neural correlates of consciousness
NIDILRR: National Institute on Disability, Independent Living, and Rehabilitation Research
PCI: Perturbational Complexity Index
PET: Positron emission tomography
rd-mPFC: Rostrodorsal medial prefrontal cortex
TMS: Transcranial magnetic stimulation
VS/UWS: Vegetative state/Unresponsive wakefulness syndrome

## Footnotes

1 Importantly, patients with DoCs might be still capable of minimal forms of behaviour, both automatic or involuntary (like in the case of Vegetative State/Unresponsive Wakefulness Syndrome) and voluntary (as in Minimally Conscious State), possibly only in terms of cerebral activity in reaction to external stimuli (as in Cognitive Motor Dissociation) or independently from them (as in non-behavioural MCS patients).

2 Peter Carruthers in his recent book *Human and Animal Mind: the Consciousness Questions Laid to Rest* (2019) outlines that the other minds problem puts back-to-front because we are born with an intrinsic attitude to attribute mind to others and on the basis of this we are able to attribute mind to ourselves. This is an interesting point, but it seems limited to the psychological level, while it does not affect the ontological and epistemological relevance of the other minds problem, i.e.: does consciousness really exist in other people? How can I know that there is a conscious activity in the brain of the person in front of me?

3 This theoretical approach to consciousness is open to the possibility of describing it as an intrinsic characteristic of the cortex and potentially other structures in the brain, and consequently to draw a distinction between consciousness and awareness, with the former covering the latter but including also unaware operations traditionally equated with unconsciousness 17. Remaining neutral with regard to this hypothesis, we will use consciousness and awareness interchangeably throughout the present paper.

4 It is theoretically possible that other modes of consciousness exist with relevant indicators, e.g. imagery and dreaming.

5 Some kind of consciousness is likely supported by other types of nervous system in non-mammalian species. The case of octopus is, for instance, highly illustrative. See [11, 55–57].

6 We would like to note that we are not denying the possibility that verbal communication pertains also to non-human animals, even if contentious and maybe more theoretical than empirical.
